# Preoperative prevalence of methicilline-resistant Staphylococcus aureus (MRSA) in non-hospitalized population in the Netherlands during a 5-year period

**DOI:** 10.1186/2047-2994-4-S1-O4

**Published:** 2015-06-16

**Authors:** V Weterings, D Arens, C Verhulst, J Kluytmans

**Affiliations:** 1 Laboratory for Microbiology and Infection Control, Amphia Hospital, Breda, the Netherlands; 2 Julius Center for Health Sciences and Primary Care, University Medical Center Utrecht, Utrecht, Netherlands

## Introduction

The MRSA Search-and-Destroy strategy relies on active screening of high risk groups. However, not all carriers belong to known risk groups and MRSA in the community is emerging also in the Netherlands.

## Objectives

We conducted a retrospective, observational study to determine the prevalence of MRSA carriers in non-hospitalized cardiothoracic, interventional cardiologic, orthopaedic and vascular patients.

## Methods

The study was performed in a large teaching hospital in the Netherlands. All samples of patients who were tested for preoperative *S. aureus* nose carriage from Mar 1, 2010 until Dec 31, 2014, were included. Nasal swabs (ESwab, Copan Diagnostics, Italy) were collected during preoperative assessments. Samples of cardiothoracic patients were tested by PCR (GeneXpert, Cepheid, CA), other samples were cultured using chromogenic agar plates. All MRSA isolates were confirmed using molecular methods. A questionnaire was conducted to ascertain potential MRSA risk factors and a linear regression analysis was used to examine trends in MRSA carriage

## Results

In total, 18,298 nasal swabs were obtained from 14,552 unique patients. In 329 swabs (1.8%) the GeneXpert gave an invalid result, therefore 17,969 swabs were included in our analysis. *S. aureus* was detected in 4604 patients (25.6% 95% CI 25.0-26.3%) of which 26 were MRSA (0.14% 95% CI 0.10-0.22%). Prevalence of MRSA carriage increased by a factor of 1.8 (from 0.12% in 2010 to 0.22% in 2014), however this increase was not significant (p=0.144 using linear regression analysis). Twelve spa types were found: 34.6% belonged to t011, 15.4% to t002, 7.7% to t015, 7.7% to t018, 7.7% to t223, and 3.8% to t024, t445, t447, t458, t688, t1154, t1784 each. Two isolates were positive for the PVL gene(7.4%). Results of the questionnaire revealed that 20/26 patients had no known risk factors for MRSA carriage (0.11% 95% CI 0.07-0.17%). In this group spa type t002 (36.4%) and t011 (36.4%) were most prevalent.

## Conclusion

This study revealed a sustained low prevalence of MRSA carriage of 0.14% in non-hospitalized patients in a large teaching hospital, over 5 years. The high prevalence of spa type t011 in patients without livestock-associated(LA) risk factors, indicates that LA-MRSA is spreading to individuals in the community

## Disclosure of interest

None declared.

